# Systematic Mutational Analysis of the Intracellular Regions of Yeast Gap1 Permease

**DOI:** 10.1371/journal.pone.0018457

**Published:** 2011-04-19

**Authors:** Ahmad Merhi, Nicolas Gérard, Elsa Lauwers, Martine Prévost, Bruno André

**Affiliations:** 1 Physiologie Moléculaire de la Cellule, Université Libre de Bruxelles, Gosselies, Belgium; 2 Structure et Fonction des Membranes Biologiques, Université Libre de Bruxelles, Bruxelles, Belgium; Centre National de la Recherche Scientifique, France

## Abstract

**Background:**

The yeast general amino acid permease Gap1 is a convenient model for studying the intracellular trafficking of membrane proteins. Present at the plasma membrane when the nitrogen source is poor, it undergoes ubiquitin-dependent endocytosis and degradation upon addition of a good nitrogen source, e.g., ammonium. It comprises 12 transmembrane domains (TM) flanked by cytosol-facing N- and C-terminal tails (NT, CT). The NT of Gap1 contains the acceptor lysines for ubiquitylation and its CT includes a sequence essential to exit from the endoplasmic reticulum (ER).

**Principal Findings:**

We used alanine-scanning mutagenesis to isolate 64 mutant Gap1 proteins altered in the NT, the CT, or one of the five TM-connecting intracellular loops (L2, -4, -6, -8 and -10). We found 17 mutations (in L2, L8, L10 and CT) impairing Gap1 exit from the ER. Of the 47 mutant proteins reaching the plasma membrane normally, two are unstable and rapidly down-regulated even when the nitrogen source is poor. Six others are totally inactive and another four, altered in a 16-amino-acid sequence in the NT, are resistant to ammonium-induced down-regulation. Finally, a mutation in L6 causes missorting of Gap1 from the secretory pathway to the vacuole. Interestingly, this direct vacuolar sorting seems to be independent of Gap1 ubiquitylation.

**Conclusions:**

This study illustrates the importance of multiple intracellular regions of Gap1 in its secretion, transport activity, and down-regulation.

## Introduction

The general amino acid permease Gap1 of *Saccharomyces cerevisiae* [1] has emerged over the last fifteen years as one of the most studied yeast plasma membrane proteins. Several aspects have been particularly well investigated, including its folding and exit from the endoplasmic reticulum (ER) [2], association with lipid rafts [3]–[5], nitrogen-regulated membrane trafficking [6], [7], post-translational modifications [8]–[10], and ability to activate signaling pathways in response to substrate loading [11]. The 602-amino-acid Gap1 protein comprises a hydrophobic core of twelve transmembrane domains (TM) flanked by intracellular N- and C-terminal tails (NT, CT) [12]. Gap1 is a member of a family of amino acid transporters highly conserved in bacteria and fungi [13] and belonging to the APC superfamily of transporters [14]. The recent elucidation of the crystal structure of two bacterial APC proteins, the AdiC arginine-agmatine antiporter [15], [16] and the ApcT broad-specificity amino-acid transporter [17], revealed that APC proteins are members of an even broader superfamily of transport proteins often named “5+5” transporters [18]. These proteins share a structural fold comprising two inversely repeated blocks of 5 TM domains and likely catalyze transmembrane solute transport via similar structural dynamics [19]. In yeast, no less than 24 APC transporters have been inventoried, most of which are amino acid permeases exhibiting various substrate-range specificities and regulations [13]. The particularity of Gap1 is that it can mediate uptake of all protein amino acids as well as citrulline, ornithine, γ-aminobutyrate (GABA), β-alanine and even D-isomers such as D-histidine. Furthermore, Gap1 shows very high affinity for most of its natural substrates, with apparent Km values in the micromolar range [20]. These properties are well suited to the physiological role of Gap1, which is synthesized and most active under conditions of poor nitrogen supply. The role of Gap1 under these conditions is to scavenge external amino acids to be used as nitrogen sources or directly as building blocks for protein synthesis. Transcription of the *GAP1* gene is promoted by two GATA-family factors, Gln3 and Gat1, which are mostly active when the nitrogen supply conditions are cell growth limiting. When cells shift to more favorable nitrogen supply conditions, the Gln3 and Gat1 factors are inhibited by the mechanisms of Nitrogen Catabolite Repression (NCR), thus causing a strong reduction of *GAP1* expression [21].

The intracellular trafficking of Gap1 has been the subject of intense investigation. Proper folding of newly synthesized Gap1 involves an integral membrane protein of the ER, Shr3 [22], which interacts with the first five TMs of the permease and prevents its aggregation [2]. Loading of Gap1 into COPII vesicles requires a di-acidic sequence in the CT that likely interacts with the Sec23/Sec24 COPII coat protein complex [23]. Normal folding of Gap1 also requires tight coupling with sphingolipid (SL) biogenesis. If Gap1 is assembled under conditions preventing ER-associated SL biogenesis, the protein exits the ER normally and reaches the plasma membrane, but is improperly folded and inactive, fails to associate with SL- and sterol-rich membrane fractions, and is subject to rapid down-regulation [5]. Under normal conditions, the Gap1 permease is subject to tight control by nitrogen, a process involving the Npr1 kinase and ubiquitin (Ub). On poor nitrogen media, e.g. when urea or proline is the sole nitrogen source, the Npr1 kinase is active and the Gap1 permease reaching the late Golgi is sorted to the plasma membrane and accumulates there in a highly active and stable form [24], [25]. When a good nitrogen source is added to the medium, Npr1 is proposed to be inactivated via the TOR signaling pathway [26]. This loss of Npr1 function triggers sorting of cell-surface Gap1 into endocytic vesicles, followed by delivery into the vacuole where it is degraded [24], [27]. Furthermore, newly synthesized Gap1 reaching the Golgi is sorted to the vacuole without passing through the plasma membrane [8], [25], [28]. Delivery of Gap1 into the vacuolar lumen involves its prior sorting into vesicles budding into the lumen of the late endosome via the multivesicular body (MVB) pathway [9], [29]. Ub is the signal triggering down-regulation of Gap1. The permease is ubiquitylated on lysines 9 or 16 (in the NT) by the Rsp5/Npi1 ubiquitin ligase [8], [27], [30]. Although linkage of a single Ub moiety to Gap1 is a sufficient signal for its internalization from the cell surface [9], the permease is modified by short Ub chains built through linkage to the K63 residue of Ub [31]. This poly-ubiquitylation was recently found to provide a specific signal for sorting into the MVB pathway [7], [9].

The membrane trafficking and regulation of Gap1 are mainly governed by interactions with several intracellular factors and by post-translational modifications of its residues exposed to the cytosol. As a further step towards getting a comprehensive view of these mechanisms, we here report the results of systematic mutagenesis of the predicted intracellular Gap1 regions.

## Materials and Methods

### Strains and growth conditions

The *S. cerevisiae* strains used in this study (Table S1) derive from the Σ1278b wild type [32]. Cells were grown at 29°C in minimal buffered medium, pH 6.1 [33]. In all experiments, the main carbon source was galactose or raffinose (3%) and a low concentration of glucose (0.3%) was also added to more readily initiate growth. Nitrogen sources were proline (10 mM), urea (10 mM), ammonium (20 to 100 mM), citrulline (1 mM), or phenylalanine (1 mM). D-histidine was added at 0.5% final concentration. The *leu2* auxotrophy was compensated by addition of leucine (0.025 mM) and the *arg5,6* auxotrophy by addition of citrulline (0.5 mM).

### Construction of plasmids

The plasmids used in this study are listed in Table S2. All derive from the centromere-based pRS416 [34] or pFL38 [35] vectors carrying the *URA3* gene. The 64 mutant *gap1* alleles were constructed by recombination in yeast between two partially overlapping PCR fragments corresponding to the 5′ and 3′ regions of the *GAL-GAP1-GFP* gene (Fig. S1). The overlapping sequence was 40 bp long and contained the sequences so as to introduce 3 or 4 consecutive alanine substitutions. The pCJ130 recipient plasmid, a pRS416 vector containing the *GAL-YCH1-GFP* gene, was linearized with *Bam*HI and treated with alkaline phosphatase. Each mutant *gap1* gene was purified by cloning into *E. coli* and verified by sequencing. The sequences of the 128 oligonucleotides used to construct the 64 mutant genes are available upon request.

### Permease assays

Gap1 activity was determined by measuring the initial uptake rate of ^14^C-labelled citrulline (20 µM) [36]. All assays were carried out in exponentially growing cells, during the state of balanced growth [37]. For the mutants of low transport activities, we measured the linear accumulation of citrulline during 60 minutes.

### Fluorescence microscopy

The steady state subcellular location of Gap1-GFP proteins was determined in cells growing exponentially in liquid galactose-proline medium. Glucose was added (final concentration: 3%) 2 hours before the cells were visualized so as to arrest Gap1-GFP neosynthesis. Labeling of the vacuolar membrane with FM4-64 was performed as described previously [29]. Cells were laid on a thin layer of 1% agarose and viewed at room temperature with a fluorescence microscope (Eclipse E600; Nikon) equipped with a 100× differential interference contrast NA 1.40 Plan-Apochromat objective (Nikon) and appropriate fluorescence light filter sets. Images were captured with a digital camera (DXM1200; Nikon) and ACT-1 acquisition software (Nikon) and processed with Photoshop CS (Adobe Systems).

### Protein extracts and Western blotting

Proteins were immunodetected in total protein extracts [30]. After transfer to a nitrocellulose membrane (Schleicher & Schüll, catalog nbr NBA085B), the proteins were probed with a monoclonal antibody raised against GFP (Roche, catalog nbr 11 814 460 001) or Pma1 [24]. Primary antibodies were detected with horseradish peroxidase–conjugated anti–mouse IgG secondary antibody (GE Healthcare, catalog nbr NA931V) followed by enhanced chemiluminescence (Roche, catalog nbr 12 015 196 001).

## Results

### Construction of 64 *gap1* alleles

**Figure 1:**
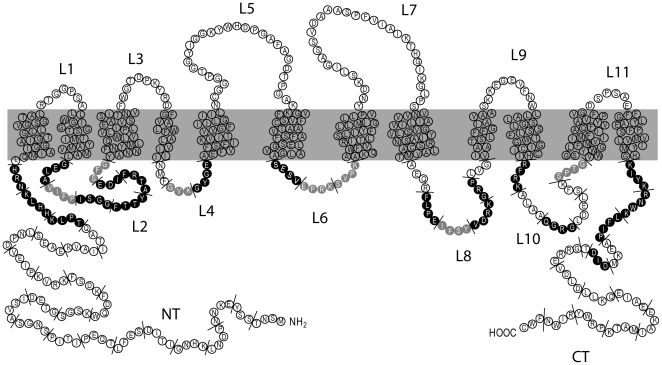
Schematic topology model of the Gap1 protein. Residues shown in black or gray in the N-terminal tail (NT), C-terminal tail (CT) and intracellular loops (L2, L4, L6, L8, L10) are those which, when replaced with alanines, cause Gap1 to be inactive or partially active, respectively (see text). The short lines delineate the mutagenized blocks of 3 or 4 amino acids. The position of the ubiquitin-acceptor lysines (K9 and K16) and of specific, more deeply analyzed, mutations is shown.

By site-directed mutagenesis we isolated a collection of 64 genes encoding Gap1 proteins having three to four consecutive amino acids replaced with alanines. The mutagenized regions cover the N-terminal tail (NT), the C-terminal tail (CT), and the five intracellular loops (L2, L4, L6, L8 and L10) connecting transmembrane (TM) domains (Table 1), i.e. all the Gap1 sequences facing the cytosol (Fig. 1). The limits of the NT, the CT, and of loops L2 to L10 were determined on the basis of sequence and structural comparisons between Gap1 and two related bacterial amino acid transporters, AdiC and ApcT (our unpublished data), whose crystal structure has been recently reported [15]–[17]. Each mutant gene was constructed by recombination in yeast between two PCR-amplified DNA fragments. The coding region of each mutant allele was cloned behind the galactose-inducible *GAL1* promoter and its 3′ end fused to the GFP coding region. Each plasmid was purified by cloning in *E. coli* and verified by sequencing before being introduced into various yeast mutants for phenotypic characterization (see Materials and methods).

**Table 1:** Functional analysis of 64 Gap1 mutants.

Mutated Gap1 region	*gap1* allele	Mutated sequence	Mutant class	Amino acid utilization	Intoxication by D-His	Localization
	*gap1-105*	2-SNT-4	F	+	+	pm
	*gap1-101*	5-SSY-7	F	+	+	pm
	*gap1-106*	8-EKNN-11	F	+	+	pm
	*gap1-107*	12-PDNL-15	F	+	+	pm
	*gap1-108*	16-KHNG-19	F	+	+	pm
	*gap1-109*	20-ITID-23	F	+	+	pm
	*gap1-110*	24-SEFL-27	F	+	+	pm
	*gap1-111*	28-TQEP-31	F	+	+	pm
	*gap1-112*	32-ITIP-35	F	+	+	pm
	*gap1-113*	36-SNGS-39	F	+	+	pm
N-terminal	*gap1-114*	40-AVSI-43	F	+	+	pm
tail	*gap1-115*	44-DETG-47	F	+	+	pm
	*gap1-116*	48-SGSK-51	F	+	+	pm
	*gap1-117*	52-WQDF55	F	+	+	pm
	*gap1-118*	56-KDSF59	F	+	+	pm
	*gap1-119*	60-KRVK-63	F	+	+	pm
	*gap1-120*	64-PIEV-67	F	+	+	pm
	*gap1-121*	68-DPNL-71	F	+	+	pm
	*gap1-122*	72-SEAE-75	F	+	+	pm
	*gap1-123*	76-KVAI-79	F	+	+	pm
	*gap1-124*	80-ITAQ-83	F	+	+	pm+v
	*gap1-125*	84-TPLK-87	NF	−	−	pm
	*gap1-126*	88-HHLK-91	NF	−	−	pm
	*gap1-127*	92-NRH-94	NF	−	−	pm
	*gap1-140*	143-GELA-146	NF	−	−	ER
	*gap1-141*	147-VIFP-150	PF	+	−	ER+pm
	*gap1-142*	151-ISGG-154	NF	−	−	pm
Loop 2	*gap1-143*	155-FTTY-158	NF	−	−	ER
	*gap1-144*	159-ATRF-162	NF	−	−	ER
	*gap1-145*	163-IDE-165	NF	−	−	ER
	*gap1-166*	166-SFG-168	PF	+	−	ER+pm
	*gap1-167*	219-NMF-221	F	+	+	pm+v
Loop 4	*gap1-168*	222-GVK-224	PF	+	−	pm
	*gap1-146*	225-GYGE-228	NF	−	−	pm
	*gap1-151*	307-SESV-310	NF	−	−	pm
Loop 6	*gap1-152*	311-EPRK-314	PF	+	−	pm+v
	*gap1-153*	315-SVPK-318	PF	+	−	pm
	*gap1-157*	405-AEQR-408	F	+	+	pm
	*gap1-158*	409-FLPE-412	NF	−	−	ER
Loop 8	*gap1-159*	413-IFSY-416	PF	+	−	ER + pm
	*gap1-160*	417-VDRK-420	NF	−	−	ER
	*gap1-169*	421-GRP-423	NF	−	−	ER
	*gap1-170*	424-LVG-426	F	+	+	pm
	*gap1-161*	473-RFRK-476	NF	−	−	ER
	*gap1-162*	475-ALAA-480	F	+	+	pm
Loop 10	*gap1-163*	481-QGRG-484	NF	−	−	ER
	*gap1-164*	485-LDEL-488	F	+	+	pm
	*gap1-165*	489-SFK-491	F	+	+	pm
	*gap1-171*	492-SPTG-495	PF	+	−	ER+pm
	*gap1-139*	548-KIYK-551	NF	−	−	ER
	*gap1-138*	552-RNWK-555	NF	−	−	ER
	*gap1-137*	556-LFIP-559	NF	−	−	ER
	*gap1-136*	560-AEKM-563	F	+	+	pm
	*gap1-104*	564-DID-566	NF	−	−	ER
	*gap1-103*	T-567	F	+	+	pm
C-terminal	*gap1-135*	568-GRRE-571	F	+	+	pm
tail	*gap1-134*	572-VDLD-575	F	+	+	pm
	*gap1-133*	576-LLKQ-579	F	+	+	pm
	*gap1-132*	580-EIAE-583	F	+	+	pm
	*gap1-131*	584-EKAI-587	F	+	+	pm
	*gap1-130*	588-MATK-591	F	+	+	pm
	*gap1-129*	592-PRWY-595	F	+	+	pm
	*gap1-128*	596-RIWN-599	F	+	+	pm
	*gap1-102*	600-FWC-602	F	+	+	pm

Strains *gap1Δ ura3* and *gap1Δ ssy1Δ ura3* transformed with the centromere-based plasmids carrying the indicated *gap1* allele were tested for growth on solid media containing citrulline or phenylalanine, respectively, as sole nitrogen source. The *gap1Δ ura3* cells were also tested for growth on a proline medium containing D-histidine. The + and − signs mean that transformed cells are able to utilize citrulline and phenylalanine (column “Amino acid utilization”) or to be intoxified by D-histidine (column “Intoxication by D-His)”). Transformed cells of the *gap1Δ ura3* strain were also grown on proline as sole nitrogen source and examined under the fluorescence microscope. The Gap1-GFP proteins were localized at the cell surface (pm, plasma membrane), vacuolar lumen (v), or endoplasmic reticulum (ER), or in several of these cell membranes. Mutant classes: F: functional, NF: non functional; PF: partially functional.

### Functionality of the mutant Gap1 permeases

**Figure 2:**
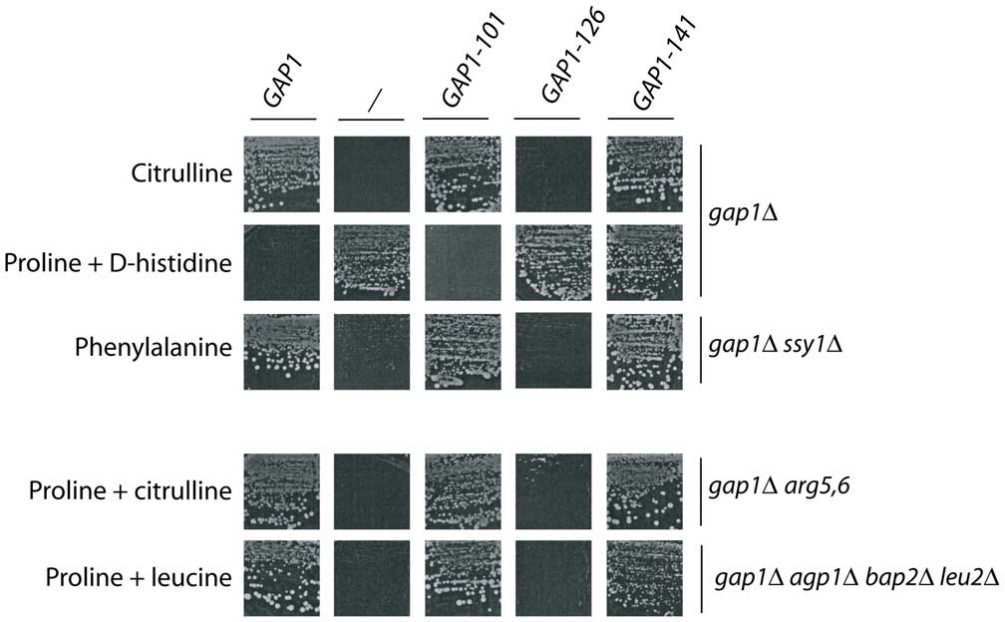
The mutant *gap1* genes confer three types of growth phenotype. Representative results of growth tests on solid minimal medium obtained with strains EK008 (*gap1Δ*), 32501d (*gap1Δ ssy1Δ*), 30788b (*gap1Δ arg5,6*), and FB097 (*gap1Δ agp1Δ bap2Δ leu2Δ*) transformed with the empty vector YCpFL38 (−), the pJOD10 (YCpGAL-GAP1-GFP) plasmid expressing the native permease (Gap1), or one of three derived plasmids encoding representative Gap1 mutants. Cells were incubated at 29°C for 4 to 7 days, depending on the nitrogen source.

Plasmids harboring the native *GAP1* allele, one of the 64 mutant *gap1* alleles, or no *GAP1* gene were introduced individually into different mutant strains suitable for assessing the functionality of Gap1 by means of growth tests on solid media. The media contained galactose as a carbon source, and their nitrogen composition varied according to the recipient strain. For instance, the *gap1Δ* strain transformed with the different plasmids was tested for growth on citrulline as sole nitrogen source (Cit medium). As uptake of this amino acid is mediated mainly by Gap1 [20], the *gap1Δ* mutant is unable to grow on this medium (Fig. 2 and Fig. S2A). The same strains were tested for growth on a medium containing proline as sole nitrogen source (Pro medium), to which the D isomer of histidine (D-His) was added or not. As uptake of proline is mediated mainly by the Put4 permease [38], strains grow normally on Pro medium even if Gap1 is inactive. As for D-His, it is toxic to cells and its uptake is solely mediated by Gap1 [20]. Hence, cells expressing the native Gap1 protein do not grow on Pro + D-His medium, whereas those expressing no Gap1 protein grow well on this medium (Fig. 2 and Fig. S2B). The plasmids were also introduced into a *gap1Δ ssy1Δ* strain. In this mutant, the Ssy1 permease-like amino-acid sensor responsible for the activation of several amino acid permeases (such as *AGP1*, *BAP2*, *GNP1*…) [39]–[41] is inactive, making Gap1 essential to growth on media containing a single amino acid such as phenylalanine (Phe) as sole nitrogen source [41] (Fig. 2 and Fig. S2C).

The results of these growth tests are presented in Table 1 and the growth phenotypes displayed by representative clones are shown in Figure 2. Among the 64 mutant proteins, 38 (59%) behaved like the native Gap1 permease and can thus be considered functional. Nineteen other mutant proteins (30%) appeared totally non-functional. The seven remaining mutant proteins (11%) conferred to cells an intermediary phenotype. Cells expressing these Gap1 forms (e.g. Gap1-141) grew normally on Cit and Phe media (suggesting functionality) and also on Pro + D-His medium (suggesting non-functionality) (Fig. 2). The transport activity of these Gap1 mutants might be low enough to protect cells against the toxic effect of D-His yet high enough to enable cells to grow normally on Cit or Phe used as sole nitrogen source. In support of this view, a five-fold increase in D-His concentration impaired growth of these Gap1-expressing clones without affecting that of cells expressing no Gap1 (data not shown). Furthermore, these *gap1* mutants proved able to fulfill the amino-acid requirements of arginine (*gap1 arg5–*6) and leucine (*gap1 agp1 bap2 leu2*) auxotrophs (Fig. 2 and data not shown), in keeping with the fact that even partially active permeases are typically able to sustain growth in this type of growth assay.

The results of these growth tests thus suggest that the mutant Gap1 proteins can be classified as functional, non-functional and partially functional (Table 1). This conclusion was supported by direct assay of Gap1 activities. The initial rate of ^14^C-citrulline uptake was measured in cells growing on urea medium, where Gap1 is normally active and stable at the plasma membrane. The functional Gap1 mutant proteins displayed uptake activities ranging from 25% to 120% of the activity of native Gap1 (Table 2 and data not shown). The ^14^C-citrulline uptake activity displayed by the non-functional Gap1 mutants did not exceed the basal level measured in the *gap1Δ* strain (less than 2% of the activity of native Gap1). The partially functional Gap1 mutants displayed very low uptake activities, ranging from 2% to 11% of the activity of native Gap1 (Table 2 and data not shown). This suggests that even very partially active Gap1 mutants can promote the utilization of amino acids as sole nitrogen source. We have also quantified the immunoblot signals of the different Gap1 mutants shown in Table 2 and found that none was present at a level less than two-fold compared to the native Gap1 protein (Fig. S3 and data not shown), i.e. their reduced or lack of activity is not due to non-expression.

**Table 2:** The mutant Gap1 proteins display variable uptake activities.

	Mutagenized region	Mutant class	Uptake activity (nmoles.min^−1^.mg prot^−1^)
None			0.4
Gap1		F	33.2
Gap1^K9,16R^	K9, K16	F	38.7
Gap1-101	NT	F	33.8
Gap1-124	NT	F	10.7
Gap1-112	NT	F	40.1
Gap1-167	L4	F	9.4
Gap1-126	NT	NF	0.6
Gap1-143	L2	NF	0.4
Gap1-146	L4	NF	0.4
Gap1-104	CT	NF	0.6
Gap1-141	L2	PF	3.6
Gap1-168	L4	PF	0.7
Gap1-152	L6	PF	3.4
Gap1-171	L10	PF	1.2

The *gap1Δ ura3* strain transformed with the centromere-based plasmids carrying the indicated *gap1* alleles were grown on a medium containing galactose as a carbon source and urea as nitrogen source. Uptake activities have been determined by assaying the rates of ^14^C-citrulline uptake (20 microM). Uptake activities correspond to averages of two independent experiments. Variations did not exceed 20%. Mutant classes: F : functional; NF : non functional; PF : partially functional. Mutated regions: NT: N-terminal tail; CT: C-terminal tail; L2, -4, -6, -10: intracellular loops 2, 4, 6 and 10.

The overall data show that mutations in Gap1 regions close to the membrane generally impair the permease function (Fig. 1). For instance, mutations in the 11 amino acids preceding the first TM abolish Gap1 activity, and the same is true for the 12 amino acids directly following the last TM. Mutations in the remaining regions of the NT and CT apparently do not impair Gap1 activity. One exception, however, is the replacement of the Asp-Ile-Asp tripeptide in the CT (Table 2). This result was expected, as this sequence is known to promote recognition of Gap1 by the Sec23/Sec24 COPII coat complex, an essential step for exit of the permease from the ER [23]. A large proportion of the mutations in intracellular loops also cause total or partial loss of Gap1 function, this being particularly true for mutations in L2 and L6.

### Subcellular locations of the mutant Gap1 permeases

**Figure 3:**
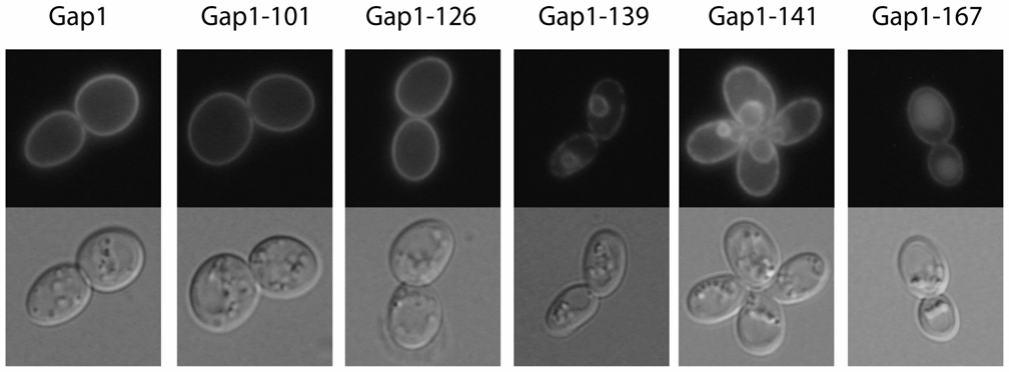
Subcellular locations of the mutant Gap1 proteins. Strain EK008 (*gap1Δ ura3*) was transformed with the pJOD10 (YCpGAL-GAP1-GFP) plasmid or with an equivalent plasmid encoding one of several representative mutant Gap1 proteins of the collection. Cells were grown on galactose-proline medium and examined by fluorescence microscopy.

Mutations causing loss of Gap1 activity might impair secretion of the permease to the plasma membrane, its stability, and/or its transport activity. To determine the subcellular locations of the mutant Gap1 proteins, *gap1Δ* mutant cells expressing a native or mutant Gap1-GFP construct were grown in liquid proline medium and examined by fluorescence microscopy. Under these conditions, the native Gap1 is known to accumulate at the plasma membrane, and its fluorescence signal was indeed present solely at the cell surface (Fig. 3). Three main subcellular location patterns were observed among the 64 mutant Gap1 proteins. A mutant representative of each pattern is presented in Figure 3.

A majority of Gap1 mutants (44 out of 64) were found at the cell surface, e.g. Gap1-101 and Gap1-126 (Fig. 3). Of these, 6 proved inactive, suggesting a loss of transport activity. These mutants were those altered in the eleven residues preceding the first TM (e.g. Gap1-126) or in intracellular loops L2, L4 and L6 (Table 1 and Fig. 3).

Seventeen other *gap1* mutants (e.g. Gap1-139 and -141) displayed a fluorescence signal around the nucleus and at the cell surface, the latter signal often being discontinuous (Fig. 3 and data not shown). This double staining is typical of proteins present in both the nuclear and cortical ER [42]. This pattern was previously observed in cells expressing the inactive Gap1-92 mutant containing a single glutamate-to-lysine substitution at position 300 and shown by membrane fractionation to be stacked in the ER [4]. The native Gap1 also displays this location pattern when expressed in cells lacking the ER-associated Shr3 chaperone [2]. These seventeen mutant Gap1 proteins thus apparently fail to properly exit the ER (Table 1). As expected, these notably include the inactive Gap1 altered in the Asp-Ile-Asp tripeptide of the CT known to promote Gap1 loading into COPII vesicles [23]. The 16 others are altered in L2, L8 or L10 and are inactive (11 mutants) or partially active (5 mutants) (Table 1). In the latter mutants, a fraction of the permease must thus be able to reach the plasma membrane and to confer some level of amino-acid uptake. Accordingly, in cells expressing these Gap1 forms (e.g. Gap1-141, Fig. 3), the fluorescence signal present at the surface is more continuous and resembles that of cells expressing native Gap1, suggesting that a fraction of the permease does indeed localize to the plasma membrane. We also carried out growth tests and localization experiments to determine whether the function or localization of the 17 ER-trapped Gap1 mutants could be rescued by overproduction of Shr3, but the results were negative (Fig. S4 and data not shown).

Finally, the fluorescence conferred by the last three Gap1 mutants (Gap1-124, -152, and -167) appeared distributed between the cell surface and the lumen of a large internal compartment corresponding to the vacuole, as confirmed by co-staining with FM4-64, a fluorescent marker accumulating at the vacuolar membrane (Fig. 3 and Fig. 5A). It thus seems that these three Gap1 mutants are targeted to the vacuole even when cells grow on a poor nitrogen source. As they are functional (Gap1-124 and Gap1-167, altered in NT and L4, respectively) or partially functional (Gap1-152, altered in L6) (Table 1), the fraction of the three proteins detectable at the cell surface is at least partially active.

### Down-regulation by ammonium of the mutant Gap1 permeases

**Figure 4:**
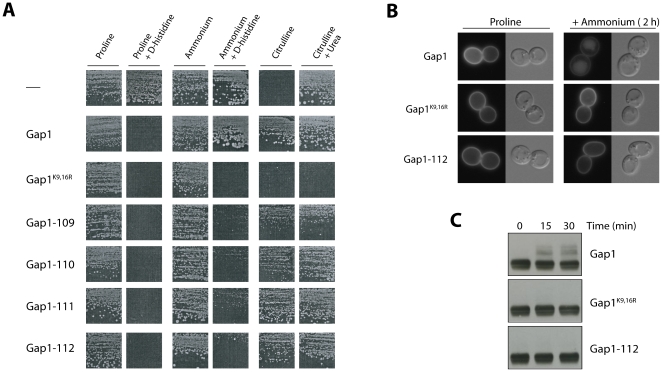
Four *gap1* mutants altered in the N-terminal tail resist ammonium-induced down-regulation. (A) Strain EK008 (*gap1Δ ura3*) transformed with the pJOD10 (YCpGAL-GAP1-GFP) plasmid expressing the native permease (Gap1) or with an equivalent plasmid expressing no Gap1 protein (−), the Gap1^K9,16R^ form resistant to ubiquitylation, or one of the indicated Gap1 mutants was tested for growth on solid medium containing the indicated nitrogenous compound(s). For each growth condition, the seven strains have been grown on the same plate. (B) Strain EK008 (*gap1Δ ura3*) transformed with the pJOD10 plasmid (Gap1) or with an equivalent plasmid encoding the Gap1^K9,16R^ or Gap1-112 mutant was grown on galactose-proline medium and examined by fluorescence microscopy before and two hours after addition of ammonium (20 mM). (C) Western blot analysis of Gap1-GFP constructs in total cell extract prepared before (t0) and several times after addition of ammonium. Strains and growth conditions were as in (B).

When ammonium (Am) is added to cells growing on a medium with urea or proline as sole nitrogen source, the Gap1 permease present at the plasma membrane is internalized by endocytosis and delivered into the lumen of the vacuole, where it is degraded. This down-regulation requires ubiquitylation on lysine 9 or lysine 16 in the NT of Gap1 [8]. Normal down-regulation of Gap1 in the presence of Am results in a growth phenotype easily discernable on a solid medium: cells expressing the *GAP1* gene under the *GAL* promoter are resistant to the toxic effect of D-His. In contrast, those expressing the Gap1^K9,16R^ form resistant to down-regulation are sensitive to D-His (Fig. 4A). Cells expressing the Gap1^K9,16R^ form are also unable to grow on Cit medium. This growth defect is not due to non-assimilation of citrulline but to some toxic effect, as shown by the inability of these cells to grow on a medium containing both citrulline and urea as nitrogen sources (Fig. 4A). To identify mutant Gap1 proteins that might be resistant to down-regulation by Am, we expressed each of the 38 active mutant proteins (i.e. those able to mediate D-His incorporation on proline medium) in *gap1Δ* cells and compared the growth phenotypes obtained on Am medium with or without D-His. Interestingly, four Gap1 mutants (Gap1-109 to -112) conferred sensitivity to D-His (Fig. 4A). Furthermore, the size of colonies on Cit medium was significantly lower for at least two of these mutants, Gap1-109 and Gap1-110 (Fig. 4A). These four Gap1 mutants are altered in a 16-amino-acid span of the NT (positions 20 to 35), close to lysines 9 and 16 (Table 1). We then used fluorescence microscopy to locate the four Gap1 mutant proteins and the native and Gap1^K9,16R^ forms used as controls (Fig. 4B and data not shown). On Pro medium, all Gap1 proteins were present at the cell surface. After addition of Am, the native Gap1 was largely targeted to the vacuolar lumen, whereas Gap1^K9,16R^ and all four Gap1 mutants stayed at the cell surface (Fig. 4B and data not shown). The 16-amino-acid region of the NT thus plays an essential role in ammonium-induced down-regulation of the Gap1 permease. An essential step of ammonium-induced endocytosis of Gap1 is its ubiquitylation on acceptor lysines K9 or K16 [8]. To determine whether the 16-amino-acid region of NT is important for ubiquitylation, we immunodetected mutant Gap1-112 in cell extracts. Native Gap1 and the Gap1^K9,16R^ mutant resistant to ubiquitylation were used as controls (Fig. 4C). In keeping with previous observations, when ammonium was added to the cells two upper bands appeared above the main signal of native Gap1 but not of Gap1^K9,16R^. These ubiquitylated forms of Gap1 were not detected for Gap1-112. In conclusion, the 16-amino acid region present in the NT of Gap1 (residues 20 to 35) appears essential to ubiquitylation of Gap1 on the neighbouring lysines 9 and 16.

### Role of ubiquitin and acceptor lysines in constitutive down-regulation of mutant Gap1 proteins

**Figure 5:**
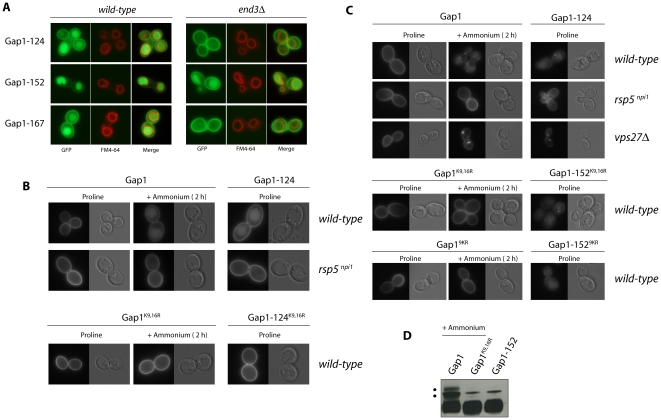
Role of ubiquitin and acceptor lysines in constitutive down-regulation of mutant Gap1 proteins. (A) Strains EK008 (*gap1Δ ura3*) and EN121 (*gap1Δ end3Δ ura3*) transformed with the pMA119 (YCpGAL-GAP1-124-GFP), pNG18 (YCpGAL-GAP1-152-GFP) or pNG47 (YCpGAL-GAP1-124-GFP) plasmids were grown on galactose-proline medium and examined by fluorescence microscopy. Cells were labeled with FM4-64 to stain the vacuolar membrane. (B) Strains EK008 (*gap1Δ ura3*) or CJ005 (*gap1Δ npi1 ura3*) were transformed with the pJOD10 (YCpGAL-GAP1-GFP) plasmid expressing the native permease (Gap1) or with an equivalent plasmid expressing the Gap1^K9,16R^ (pCJ038), Gap1-124 (pMA19) or Gap1-124^K9,16R^ (pMA142) variants. Cells were grown on galactose-proline medium and ammonium (20 mM) was eventually added for two hours together with glucose. Cells were examined by fluorescence microscopy. (C) Strains EK008 (*gap1Δ ura3*), CJ005 (*gap1Δ npi1 ura3*) and EL002 (*gap1Δ vps27Δ ura3*) were transformed with the pJOD10 plasmid (YCpGAL-GAP1-GFP) expressing the native Gap1 or with an equivalent plasmid expressing the Gap1^K9,16R^ (pCJ038), Gap1^9KR^ (pMA150) Gap1-152 (pNG18), Gap1-152^K9,16R^ (pMA145), or Gap1-152^9KR^ (pMA151) variants. Cells were grown on medium containing raffinose as carbon source and proline or ammonium (100 mM) as the sole nitrogen source. Synthesis of the Gap1 variants was induced for one hour by adding galactose. Glucose was then added for one additional hour to repress synthesis of the Gap1 proteins. Cells were then examined by fluorescence microscopy. (D) Strain EL002 (*gap1Δ vps27Δ ura3*) transformed with the pJOD10 plasmid (Gap1) or with an equivalent plasmid encoding the Gap1^K9,16R^ (pCJ038) or Gap1-152 (pNG18) mutants was grown on minimal medium containing raffinose as carbon source and proline or ammonium (100 mM) as the sole nitrogen source. Synthesis of the Gap1 variants was induced by adding galactose for one hour and stopped by adding glucose. One hour after glucose addition, cell extracts were prepared and blotted with anti-GFP antibody. The marked upper band (*) corresponds to an unspecific signal. It is well visible here because immunoblots have to be exposed for a longer time when the synthesis of Gap1 is induced for only one hour.

As illustrated above, the Gap1-152, -167, -124 mutants undergo constitutive down-regulation, i.e. they are targeted to the vacuole even on poor nitrogen media (Fig. 5A). These three Gap1 mutants were introduced into a *gap1Δ* strain containing an *end3Δ* mutation impairing endocytosis [43]. Under these conditions, the Gap1-124 and Gap1-167 mutant proteins were found only at the cell surface (Fig. 4A), indicating that they reach the plasma membrane normally but enter the endocytic pathway to be delivered to the vacuolar lumen. In contrast, the Gap1-152 protein is distributed between the cell surface and the vacuolar lumen even in the *end3* mutant (Fig. 5A). This indicates that a fraction of the neosynthesized Gap1-152 is deviated from the secretory pathway to the vacuole without passing through the plasma membrane. Interestingly, this is the typical behavior of Gap1 neosynthesized in mutants lacking the Npr1 kinase [24].

We next sought to investigate the role of Ub in the down-regulation of the Gap1-124 and Gap1-152 mutants. The Gap1-124 protein with additional K9R and K16R substitutions is stabilized at the plasma membrane (Fig. 5B). The same is true when Gap1-124 is expressed in the *npi1* mutant strain. In this strain, the Rsp5 ubiquitin ligase responsible for Gap1 ubiquitylation is expressed at a much reduced level and this impairs Gap1 ubiquitylation [27], [30]. As expected, a similar stabilization has been observed in *npi1* mutant cells expressing native Gap1 and to which ammonium has been added (Fig. 5B). Hence, down-regulation from the cell surface of Gap1-124 seems to rely on mechanisms similar to those induced by good nitrogen sources.

We next analyzed Gap1-152 and compared it to native Gap1 newly synthesized in the presence of ammonium, conditions causing the permease to be directly sorted from the Golgi to the vacuole (Fig. 5C). In the *npi1* mutant, native Gap1 synthesized on ammonium is redirected to the cell surface, in keeping with previous observations [8]. The Gap1-152 synthesized in the *npi1* mutant grown on proline medium is distributed between the cell surface and the vacuolar membrane, showing that a normal high level of the Rsp5 ubiquitin ligase is needed to promote constitutive delivery of Gap1-152 to the vacuolar lumen (Fig. 5C). We then examined the influence of K9R and K16R mutations. As expected, the Gap1^K9,16R^ permease neosynthesized in the presence of ammonium was redirected to the plasma membrane. In contrast, the Gap1-152^K9,16R^ mutant was still delivered to the vacuolar lumen in proline-grown cells (Fig. 5C). We thus reasoned that the direct vacuolar sorting of Gap1-152 involves its ubiquitylation on alternative lysines. In a previous study, we reported that a native Gap1 newly synthesized in the absence of sphingolipid biogenesis is misfolded, inactive and constitutively targeted for degradation after having reached the plasma membrane. The Gap1^K9,16R^ mutant was still down-regulated in this mutant context and only a Gap1 mutant with all nine lysines present in NT replaced with arginine turned out to be protected against degradation and stabilized at the plasma membrane [5]. Hence, under particular conditions, Rsp5-dependent down-regulation of Gap1 relies on lysines other than K9 and K16. We thus combined Gap1-152 to the nine K-to-R substitutions in the NT. Remarkably, this Gap1-152^9KR^ form was also delivered to the vacuolar lumen on proline medium (Fig. 5C), and the same was true in the *end3* mutant (data not shown). These observations prompted us to determine whether Gap1-152 undergoes ubiquitylation. For this, we used a *vps27Δ* mutant in which Vps27, a key protein of the multivesicular body (MVB) sorting machinery, is lacking [44]. This mutation impairs MVB sorting and causes a strong enlargement of the late endosome which is then referred to as the class E compartment. In such *vps* mutants, Gap1 en route to the vacuole is typically trapped in the class E compartment in a ubiquitylated form [29]. The results of Fig. 5C show that native Gap1 newly synthesized in the presence of ammonium indeed accumulates in the class E compartment of *vps27Δ* cells. Furthermore, native Gap1 in the class E compartment is ubiquitylated as upper bands (which are not detected when using the Gap1^K9,16R^ mutant) are readily detected above the main Gap1 signal in immunoblots (Fig. 5D). The Gap1-152 newly synthesized on proline medium also accumulates in the class E compartment of *vps27Δ* mutant cells (Fig. 5C), indicating that its delivery to the vacuole involves its prior sorting into the MVB pathway, as expected. Remarkably, ubiquitylated forms of Gap1-152 could not be detected in this strain (Fig. 5D), even when the immunoblots were overexposed. Furthermore, ubiquitylated forms of Gap1-152 were also undetectable under steady state conditions (growth on proline medium), conditions under which ubiquitylation of native Gap1 is readily visible after addition of ammonium to the cells (Fig. S6). These data, together with the observation that the lysine-to-arginine substitutions in Gap1-152 does not impair its vacuolar sorting, raises the interesting possibility that Gap1-152 does not need to be ubiquitylated to be sorted to the vacuole.

## Discussion

We here report the generation (by alanine-scanning mutagenesis) and preliminary phenotypic characterization of 64 mutations affecting the yeast Gap1 permease. At the protein level, each mutation involves the replacement of three to four consecutive residues with alanine. Together these substitutions cover the regions of the permease that are exposed to the cytosol: NT, CT, and L2 to L10. This collection provides an invaluable tool for investigating aspects of Gap1 in which many research teams are interested: its intracellular trafficking, transport function, and ability to activate signaling pathways.

All of the Gap1 forms altered in the NT appear to be properly targeted to the cell surface, indicating that this permease region does not play an important role in secretion. The mutants altered in the first 83 N-terminal residues are active, whereas those mutated in the last 11 residues are inactive (Fig. 1). The latter region, close to the membrane, thus seems important for the transport activity of the permease. It might notably contribute to the conformational changes associated with amino acid transport. When the Ile-Thr-Ala-Gln sequence just preceding this region is mutated, the permease (Gap1-124) reaches the plasma membrane normally and is active, but it is rapidly sorted into the endocytic pathway and delivered into the vacuole in a manner dependent on the Rsp5 ubiquitin ligase and the K9 and K16 acceptor lysines of Gap1 ubiquitylation. Mutations altering this sequence might mimic a conformational change of the permease that normally promotes its down-regulation. They might also disturb an interaction with a protein or with lipids that would stabilize Gap1 at the plasma membrane. Mutagenesis of the NT also unraveled a region spanning positions 20 to 35 that is as important as the K9 and K16 acceptor lysines for normal Am-induced ubiquitylation and down-regulation of Gap1. Our current experiments aim at determining whether this region of the NT corresponds to the interaction surface for factors promoting proper ubiquitylation of Gap1.

In contrast to the NT, the CT of Gap1 plays an important role in secretion to the plasma membrane. It notably contains the di-acidic sequence Asp-Ile-Asp previously reported to promote loading of the permease into COPII vesicles [23]. Our results confirm that this sequence is crucial for Gap1 to exit the ER. We further show that mutations in a 12-amino-acid region preceding this peptide in the CT and directly following the last TM also cause retention of Gap1 in the ER. This retention could result from improper presentation of the di-acidic motif and/or from misfolding of the mutant protein. Mutations in more C-terminally located parts of the CT did not alter the activity, secretion, or down-regulation of the permease. In particular, these processes were unaltered upon mutation of the last three residues, Phe-Trp-Cys, highly conserved among yeast amino acid permeases [45]. A recent paper reports that the cysteine of this terminal tripeptide is palmitoylated by the Pfa4 palmitoyltransferase [10]. As we detected no phenotype for the Gap1-102 mutant in which the tripeptide is mutated, the role of Gap1 palmitoylation remains unclear. We previously reported that the CT of Gap1 contains a predicted amphipathic helix including a di-leucine motif (residues 575–576). When this di-leucine or neighboring amino acids are mutated, Gap1 down-regulation by Am is impaired [45]. Despite this, the Gap1-133 mutant where the LLKQ sequence has been mutagenized did not display increased sensitivity to D-His on Am medium. This is probably due to the fact that mutation of this di-leucine affords less protection against Am-induced down-regulation than mutation of the K9 and K16 residues or of the above-mentioned 16-amino-acid region (our unpublished observations).

Among the 25 mutant proteins featuring amino acid substitutions in the intracellular loops, about half were found to be stacked in the ER. Most of them are altered in L2, L8, or L10. Four of them showed weak transport activity, suggesting that a small fraction of the synthesized protein escapes the ER and reaches the plasma membrane. Although an active role of these sequences in loading the permease into COPII vesicles is envisageable, we think they are more likely to be stacked in the ER because of improper folding. Active retention of misfolded proteins in the ER is a well-known quality control system of the secretory pathway, which often promotes the ubiquitin- and proteasome-dependent degradation of abnormal cargoes via the endoplasmic-reticulum-associated degradation (ERAD) pathway [46], [47]. For instance, ERAD of Gap1 occurs when the Shr3 chaperone is deficient [2]. It will thus be interesting to determine whether the Gap1 mutants blocked in the ER are substrates of the ERAD pathway, and if so, which lysine(s) are targets for ubiquitylation.

Eleven Gap1 mutants altered in L2 to L10 reach the plasma membrane normally. Among these, about half display no or very low activity and the others, although they behave like native Gap1 in growth tests, display significantly reduced activities. This suggests that the intracellular loops are particularly important for the transport function of the permease. For instance, these regions might be important for the dynamic properties of the permease [19]. Among the Gap1 mutants altered in intracellular loops and which are active, Gap1-167 is unstable at the plasma membrane, just like Gap1-124. Further experiments are needed to determine why this Gap1 mutant is constitutively sorted in the endocytic pathway.

The Gap1-152 mutant altered in a Glu-Pro-Arg-Lys sequence in L6 is largely sorted from the secretory pathway and delivered to the vacuolar lumen via the multivesicular body pathway. This direct vacuolar sorting is normally observed for native Gap1 when the Npr1 kinase is inactive or when a good nitrogen source is available, causing Npr1 inactivation via the TOR signaling pathway [8], [25]. Under these conditions, Gap1 reaching the late Golgi is ubiquitylated on its lysines K9 and K16 by Rsp5. This ubiquitylation is not needed for Golgi-to-endosome transport of Gap1, but is essential to its subsequent sorting into the MVB pathway [9]. Remarkably, the delivery of Gap1-152 mutant from the secretory pathway to the vacuole seems to rely on different mechanisms. We indeed observed that the vacuolar sorting of Gap1-152 is not impaired by the replacement of its K9 and K16 with arginine, and the same were true when the nine lysines present in its NT were mutated. Furthermore, we failed to detect any ubiquitylation of Gap1-152. Although these observations suggest that vacuolar sorting of Gap1-152 is independent of its ubiquitylation, Gap1-152 was missorted to the vacuolar membrane in the *npi1* mutant in which the Rsp5 ubiquitin ligase is expressed at an insufficient level. To account for these observations, we propose that Gap1-152 displays abnormal structural features leading to its recognition by a quality control system operating downstream from the ER, e.g. at a late Golgi level, as illustrated by the case of mutant forms of the Pma1 ATPase [46]. This quality control might promote Rsp5-dependent ubiquitylation of Gap1 on other lysines than those present in the NT, and this ubiquitylation might for some reasons be less stable and barely detectable under our experimental conditions. Alternatively, Gap1-152 ubiquitylation might be dispensable for its down-regulation. For instance, the quality control system might involve recognition of Gap1-152 by another factor whose Rsp5-dependent ubiquitylation would promote its MVB sorting together with the associated Gap1-152 protein. Our current experiments aim at testing the validity of these models.

In conclusion, this study describes the first systematic mutational analysis of the intracellular regions of a transmembrane transport protein, the yeast Gap1 permease. The phenotypic analysis of the 64 mutants opens new prospects for studying various aspects of Gap1 function and trafficking. It will be useful to introduce single-amino-acid substitutions within each mutagenized sequence of interest and to extend this type of systematic mutagenesis to other amino acid permeases or other transporters for data comparisons.

## Supporting Information

Figure S1 **The site-directed mutagenesis strategy used to isolate the 64 mutant** ***gap1*** **alleles.** Two PCR fragments containing the mutation were introduced into the *gap1Δ* yeast strain together with the linearized pCJ130 (*GAL-YCH1-GFP*, centromeric *URA3*) plasmid. Yeast transformants were selected for the Ura3^+^ phenotype. The plasmids generated by recombination were purified by cloning into *E. coli* and sequenced.
(TIF)

Figure S2 **Growth phenotype conferred by the 64 mutant** ***gap1*** **alleles.** (A) Strain EK008 (*gap1Δ ura3*) transformed with the pJOD10 (YCpGAL-GAP1-GFP) plasmid expressing the native permease (Gap1) or with equivalent plasmids expressing none Gap1 protein (−), the Gap1^K9,16R^ form resistant to ubiquitylation, or one of the 64 Gap1 mutants were tested for growth on a solid medium containing citrulline as sole nitrogen source. (B) Same as in (A) except that the medium contained proline as nitrogen source to which D-histidine was added or not. (C) Same as in (A) except that the strain was 32501d (*gap1Δ ssy1Δ ura3*) and the medium contained proline or phenylalanine as sole nitrogen source. Cells were grown at 29°C for 4 to 7 days according to nitrogen medium.
(TIF)

Figure S3 **Reduced or lack of activity of several tested Gap1 mutants is not due to their non-expression.** Strain EK008 (*gap1Δ ura3*) transformed with the pJOD10 (YCpGAL-GAP1-GFP) plasmid expressing the native permease (Gap1) or with equivalent plasmids expressing the Gap1^K9,16R^ form resistant to ubiquitylation, or one of several Gap1 mutants (those analyzed in Table 2) were grown on urea as sole nitrogen source. Cell extracts were prepared and immunoblotted using antibodies against GFP or Pma1 (used as a loading control). The normalized intensity of signals were: 1 (Gap1), 1.6 (Gap1^K9,16R^), 2.4 (Gap1-101), 1 (Gap1-124), 2.4 (Gap1-112), 1.4 (Gap1-167), 1.1 (Gap1-126), 0.7 (Gap1-143), 1.6 (Gap1-146), 0.6 (Gap1-104), 0.7 (Gap1-141), 0.9 (Gap1-168), 0.5 (Gap1-152), 1.1 (Gap1-171).
(TIF)

Figure S4 **Overproduction of Shr3 does not suppress the growth phenotype of Gap1 mutants trapped in the ER.** Strain ME042 (*GAL1-SHR3 gap1Δ ura3*) transformed with the pJOD10 (YCpGAL-GAP1-GFP) plasmid expressing the native permease (Gap1) or with equivalent plasmids expressing none Gap1 protein (empty vector), or one of the Gap1 mutants trapped in the ER (see text), were tested for growth on a solid medium containing galactose as a carbon source and proline as nitrogen source to which D-histidine was added or not.
(TIF)

Figure S5 **Gap1-152 does not seem to be ubiquitylated.** Strain EL002 (*gap1Δ vps27Δ ura3*) transformed with the pJOD10 plasmid (Gap1) or with an equivalent plasmid encoding the Gap1^K9,16R^ (pCJ038) or Gap1-152 (pNG18) variants was grown on galactose-proline medium. Ammonium (20 mM) was added for two hours to the cells expressing Gap1 or Gap1^K9,16R^. Cell extracts were then prepared and blotted with anti-GFP antibody.
(TIF)

Table S1 **Strains used in this study.**
(DOC)

Table S2 **Plasmids used in this study.**
(DOC)

## References

[1] Jauniaux JC, Grenson M (1990). GAP1, the general amino acid permease gene of *Saccharomyces cerevisiae*. Nucleotide sequence, protein similarity with the other bakers yeast amino acid permeases, and nitrogen catabolite repression.. Eur J Biochem.

[2] Kota J, Gilstring CF, Ljungdahl PO (2007). Membrane chaperone Shr3 assists in folding amino acid permeases preventing precocious ERAD.. J Cell Biol.

[3] Bagnat M, Simons K (2002). Cell surface polarization during yeast mating.. Proc Natl Acad Sci USA.

[4] Lauwers E, André B (2006). Association of yeast transporters with detergent-resistant membranes correlates with their cell-surface location.. Traffic.

[5] Lauwers E, Grossmann G, André B (2007). Evidence for Coupled Biogenesis of Yeast Gap1 Permease and Sphingolipids: Essential Role in Transport Activity and Normal Control by Ubiquitination.. Mol Biol Cell.

[6] Magasanik B, Kaiser CA (2002). Nitrogen regulation in *Saccharomyces cerevisiae*.. Gene.

[7] Lauwers E, Erpapazoglou Z, Haguenauer-Tsapis R, André B (2010). The ubiquitin code of yeast permease trafficking.. Trends Cell Biol.

[8] Soetens O, De Craene J-O, André B (2001). Ubiquitin is required for sorting to the vacuole of the yeast general amino acid permease, Gap1.. J Biol Chem.

[9] Lauwers E, Jacob C, André B (2009). K63-linked ubiquitin chains as a specific signal for protein sorting into the multivesicular body pathway.. Journal of Cell Biology.

[10] Roth AF, Wan J, Bailey AO, Sun B, Kuchar JA (2006). Global analysis of protein palmitoylation in yeast.. Cell.

[11] Van Zeebroeck G, Bonini BM, Versele M, Thevelein JM (2009). Transport and signaling via the amino acid binding site of the yeast Gap1 amino acid transceptor.. Nat Chem Biol.

[12] Gilstring CF, Ljungdahl PO (2000). A method for determining the *in vivo* topology of yeast polytopic membrane proteins demonstrates that Gap1p fully integrates into the membrane independently of Shr3p.. The Journal of Biological Chemistry.

[13] Van Belle D, André B (2001). A genomic view of yeast membrane transporters.. Current Opinion in Cell Biology.

[14] Jack DL, Paulsen IT, Saier MH (2000). The amino acid/polyamine/organocation (APC) superfamily of transporters specific for amino acids, polyamines and organocations.. Microbiology.

[15] Gao X, Lu F, Zhou L, Dang S, Sun L, Li X, Wang J, Shi Y (2009). Structure and mechanism of an amino acid antiporter.. Science.

[16] Fang Y, Jayaram H, Shane T, Kolmakova-Partensky L, Wu F (2009). Structure of a prokaryotic virtual proton pump at 3.2 A resolution.. Nature.

[17] Shaffer PL, Goehring A, Shankaranarayanan A, Gouaux E (2009). Structure and mechanism of a Na+-independent amino acid transporter.. Science.

[18] Krishnamurthy H, Piscitelli CL, Gouaux E (2009). Unlocking the molecular secrets of sodium-coupled transporters.. Nature.

[19] Diallinas G (2008). Biochemistry. An almost-complete movie.. Science.

[20] Grenson M, Hou C, Crabeel M (1970). Multiplicity of the amino acid permeases in *Saccharomyces cerevisiae*. IV. Evidence for a general amino acid permease.. Journal of Bacteriology.

[21] Cooper TG (2002). Transmitting the signal of excess nitrogen in *Saccharomyces cerevisiae* from the Tor proteins to the GATA factors: connecting the dots.. FEMS Microbiol Rev.

[22] Ljungdahl PO, Gimeno CJ, Styles CA, Fink GR (1992). SHR3 : a novel component of the secretory pathway specifically required for localization of amino acid permeases in yeast.. Cell.

[23] Malkus P, Jiang F, Schekman R (2002). Concentrative sorting of secretory cargo proteins into COPII-coated vesicles.. J Cell Biol.

[24] De Craene J-O, Soetens O, André B (2001). The Npr1 kinase controls biosynthetic and endocytic sorting of the yeast Gap1 permease.. J Biol Chem.

[25] Roberg KJ, Rowley N, Kaiser CA (1997). Physiological regulation of membrane protein sorting late in the secretory pathway of *Saccharomyces cerevisiae*.. J Cell Biol.

[26] Schmidt A, Beck T, Koller A, Kunz J, Hall M (1998). The TOR nutrient signaling pathway phosphorylates NPR and inhibits turnover of the tryptophane permease.. The European Molecular Biology Organization Journal.

[27] Springael J-Y, André B (1998). Nitrogen-regulated ubiquitylation of the Gap1 permease of *Saccharomyces cerevisiae*.. Mol Biol Cell.

[28] Helliwell SB, Losko S, Kaiser CA (2001). Components of a ubiquitin ligase complex specify polyubiquitylation and intracellular trafficking of the general amino acid permease.. J Cell Biol.

[29] Nikko E, Marini A-M, André B (2003). Permease recycling and ubiquitination status reveal a particular role for Bro1 in the multivesicular body pathway.. J Biol Chem.

[30] Hein C, Springael J-Y, Volland C, Haguenauer-Tsapis R, André B (1995). *NPI1*, an essential gene involved in induced degradation of Gap1 and Fur4 permeases, encodes the Rsp5 ubiquitin-protein ligase.. Mol Microbiol.

[31] Springael J-Y, Galan J-M, Haguenauer-Tsapis R, André B (1999). NH_4_
^+^-induced down-regulation of the *Saccharomyces cerevisiae* Gap1p permease involves its ubiquitination with lysine-63-linked chains.. J Cell Sci.

[32] Béchet J, Grenson M, Wiame JM (1970). Mutations affecting the repressibility of arginine biosynthetic enzymes in *Saccharomyces cerevisiae*.. Eur J Biochem.

[33] Jacobs P, Jauniaux JC, Grenson M (1980). A cis-dominant regulatory mutation linked to the argB-argC gene cluster in *Saccharomyces cerevisiae*.. J Mol Biol.

[34] Sikorski RS, Hieter P (1989). A system of shuttle vectors and yeast host strains designed for efficient manipulation of DNA in *Saccharomyces cerevisiae*.. Genetics.

[35] Bonneaud N, Ozier-Kalogeropoulos O, Li GY, Labouesse M, Minvielle-Sebastia L (1991). A family of low and high copy replicative, integrative and single-stranded S. cerevisiae/E. coli shuttle vectors.. Yeast.

[36] Grenson M, Mousset M, Wiame JM, Bechet J (1966). Multiplicity of the amino acid permeases in *Saccharomyces cerevisiae*. I. Evidence for a specific arginine-transporting system.. Biochim Biophys Acta.

[37] Wiame JM, Grenson M, Arst HN (1985). Nitrogen catabolite repression in yeasts and filamentous fungi.. Adv Microb Physiol.

[38] Lasko PF, Brandriss MC (1981). Proline transport in *Saccharomyces cerevisiae*.. J Bacteriol.

[39] Didion T, Regenberg B, Jorgensen MU, Kielland-Brandt MC, Andersen HA (1998). The permease homologue Ssy1p controls the expression of amino acid and peptide transporter genes in *Saccharomyces cerevisiae*.. Mol Microbiol.

[40] Klasson H, Fink GR, Ljungdahl PO (1999). Ssy1p and Ptr3p are plasma membrane components of a yeast system that senses extracellular amino acids.. Mol Cell Biol.

[41] Iraqui I, Vissers S, Bernard F, De Craene JO, Boles E (1999). Amino acid signaling in Saccharomyces cerevisie : a permease-like sensor of external amino acids and F-Box protein Grr1p are required for transcriptional induction of the AGP1 gene, which encodes a broad-specificity amino acid permease.. Mol Cell Biol.

[42] Lowe M, Barr FA (2007). Inheritance and biogenesis of organelles in the secretory pathway.. Nat Rev Mol Cell Biol.

[43] Bénédetti H, Raths S, Crausaz F, Riezman H (1994). The END3 gene encodes a protein that is required for the internalization step of endocytosis and for actin cytoskeleton organization in yeast.. Mol Biol Cell.

[44] Shih SC, Katzmann DJ, Schnell JD, Sutanto M, Emr SD (2002). Epsins and Vps27p/Hrs contain ubiquitin-binding domains that function in receptor endocytosis.. Nat Cell Biol.

[45] Hein C, André B (1997). A C-terminal di-leucine motif nearby sequences are required for NH_4_
^+^-induced inactivation and degradation of the general amino acid permease, Gap1p, of *Saccharomyces cerevisiae*.. Mol Microbiol.

[46] Arvan P, Zhao X, Ramos-Castaneda J, Chang A (2002). Secretory pathway quality control operating in Golgi, plasmalemmal, and endosomal systems.. Traffic.

[47] Hirsch C, Gauss R, Horn SC, Neuber O, Sommer T (2009). The ubiquitylation machinery of the endoplasmic reticulum.. Nature.

