# Supplementary figures and images for: Systematic Mutational Analysis of the Intracellular Regions of Yeast Gap1 Permease
Source: PLoS One. 2011 Apr 19;6(4):e18457. doi: 10.1371/journal.pone.0018457 (PMC3079708; doi:10.1371/journal.pone.0018457)

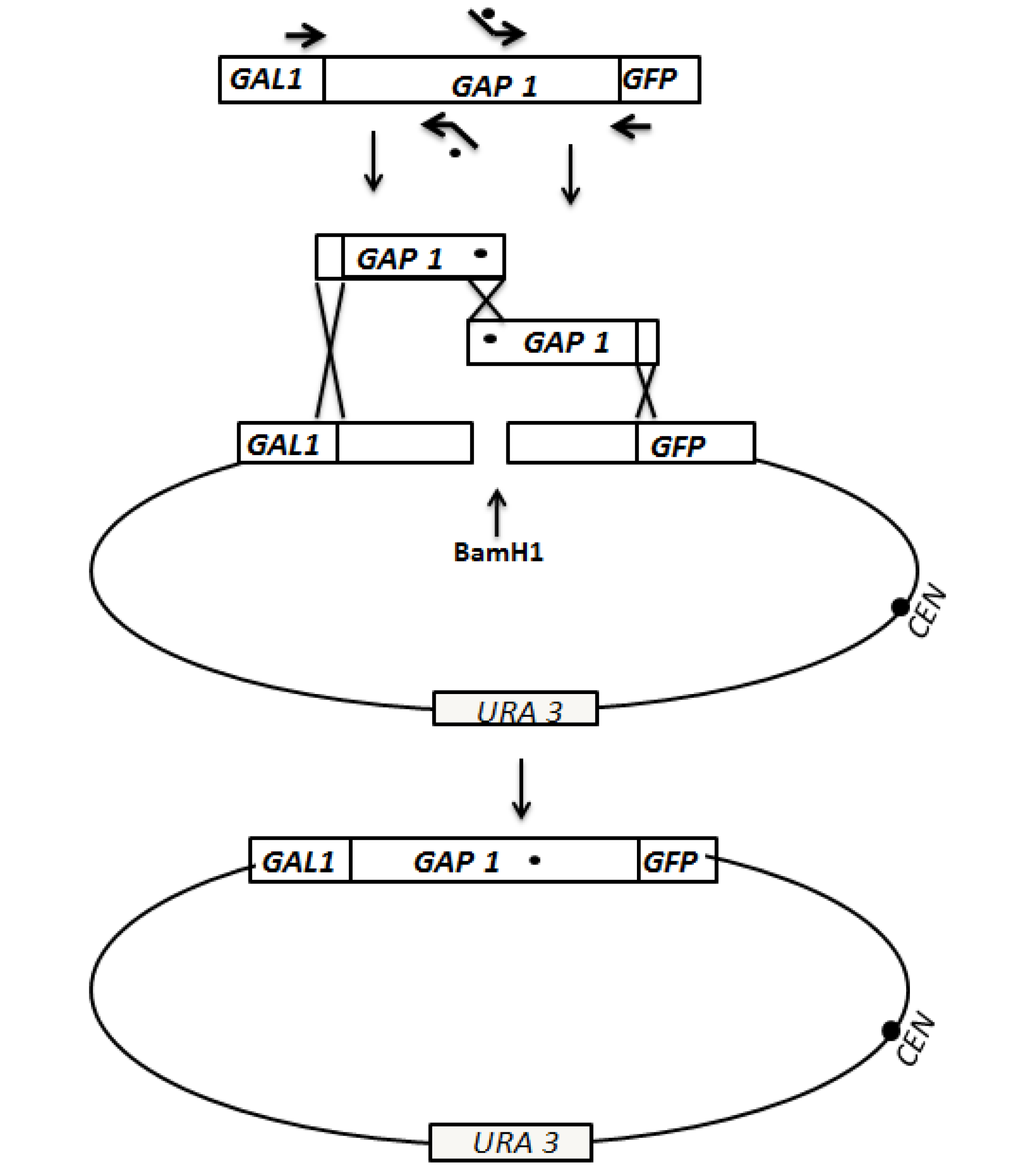

Supplement: Figure S1 — The site-directed mutagenesis strategy used to isolate the 64 mutant gap1 alleles. Two PCR fragments containing the mutation were introduced into the gap1Δ yeast strain together with the linearized pCJ130 (GAL-YCH1-GFP, centromeric URA3) plasmid. Yeast transformants were selected for the Ura3+ phenotype. The plasmids generated by recombination were purified by cloning into E. coli and sequenced. (TIF) [file pone.0018457.s001.tif]

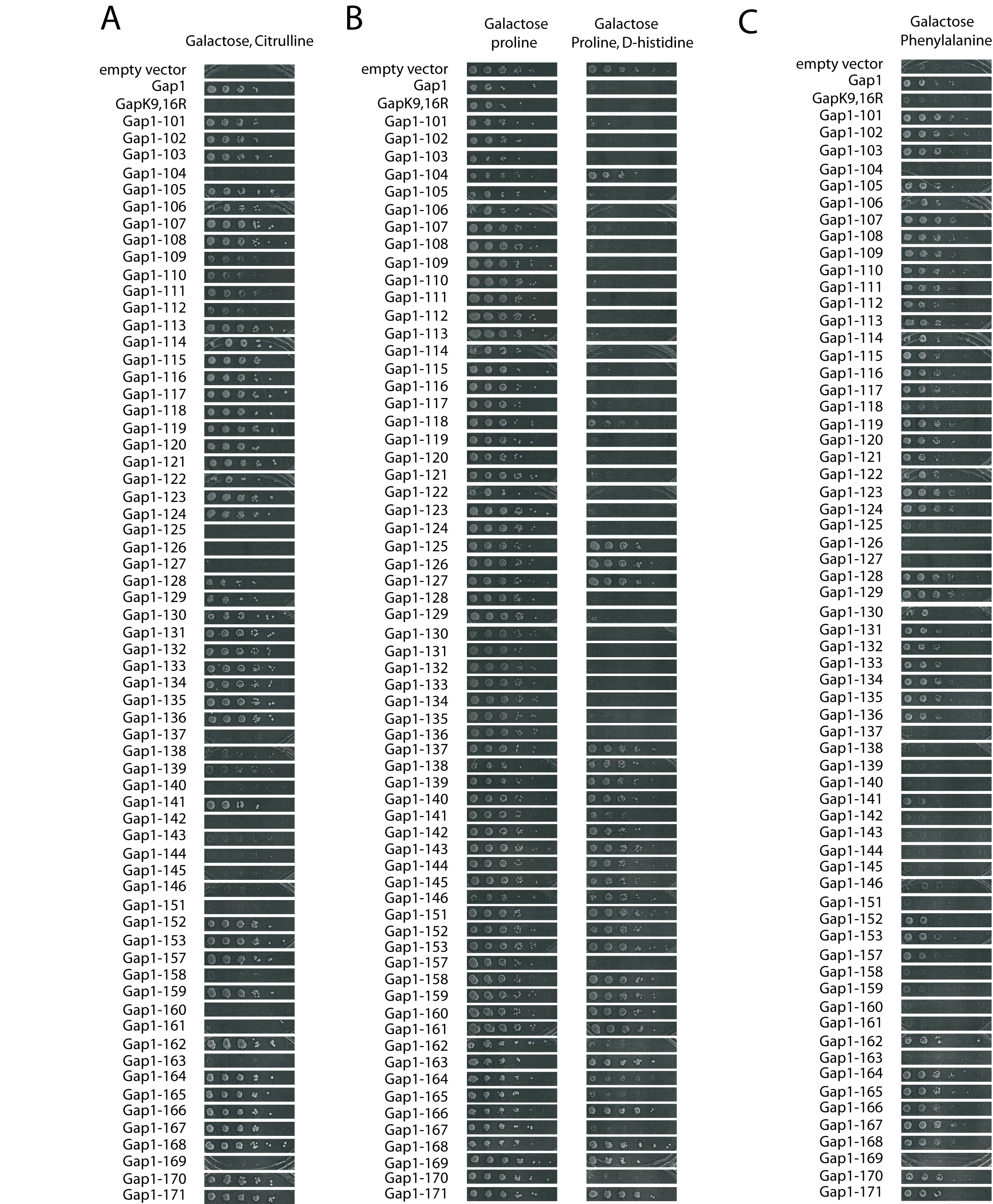

Supplement: Figure S2 — Growth phenotype conferred by the 64 mutant gap1 alleles. (A) Strain EK008 (gap1Δ ura3) transformed with the pJOD10 (YCpGAL-GAP1-GFP) plasmid expressing the native permease (Gap1) or with equivalent plasmids expressing none Gap1 protein (−), the Gap1K9,16R form resistant to ubiquitylation, or one of the 64 Gap1 mutants were tested for growth on a solid medium containing citrulline as sole nitrogen source. (B) Same as in (A) except that the medium contained proline as nitrogen source to which D-histidine was added or not. (C) Same as in (A) except that the strain was 32501d (gap1Δ ssy1Δ ura3) and the medium contained proline or phenylalanine as sole nitrogen source. Cells were grown at 29°C for 4 to 7 days according to nitrogen medium. (TIF) [file pone.0018457.s002.tif]

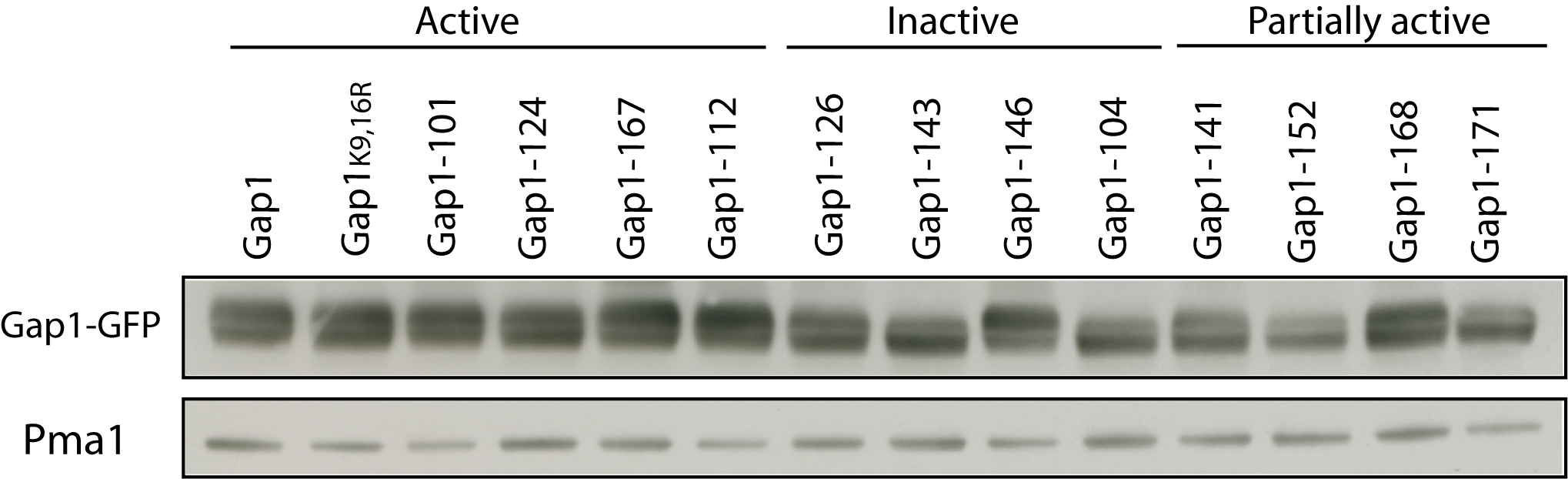

Supplement: Figure S3 — Reduced or lack of activity of several tested Gap1 mutants is not due to their non-expression. Strain EK008 (gap1Δ ura3) transformed with the pJOD10 (YCpGAL-GAP1-GFP) plasmid expressing the native permease (Gap1) or with equivalent plasmids expressing the Gap1K9,16R form resistant to ubiquitylation, or one of several Gap1 mutants (those analyzed in Table 2) were grown on urea as sole nitrogen source. Cell extracts were prepared and immunoblotted using antibodies against GFP or Pma1 (used as a loading control). The normalized intensity of signals were: 1 (Gap1), 1.6 (Gap1K9,16R), 2.4 (Gap1-101), 1 (Gap1-124), 2.4 (Gap1-112), 1.4 (Gap1-167), 1.1 (Gap1-126), 0.7 (Gap1-143), 1.6 (Gap1-146), 0.6 (Gap1-104), 0.7 (Gap1-141), 0.9 (Gap1-168), 0.5 (Gap1-152), 1.1 (Gap1-171). (TIF) [file pone.0018457.s003.tif]

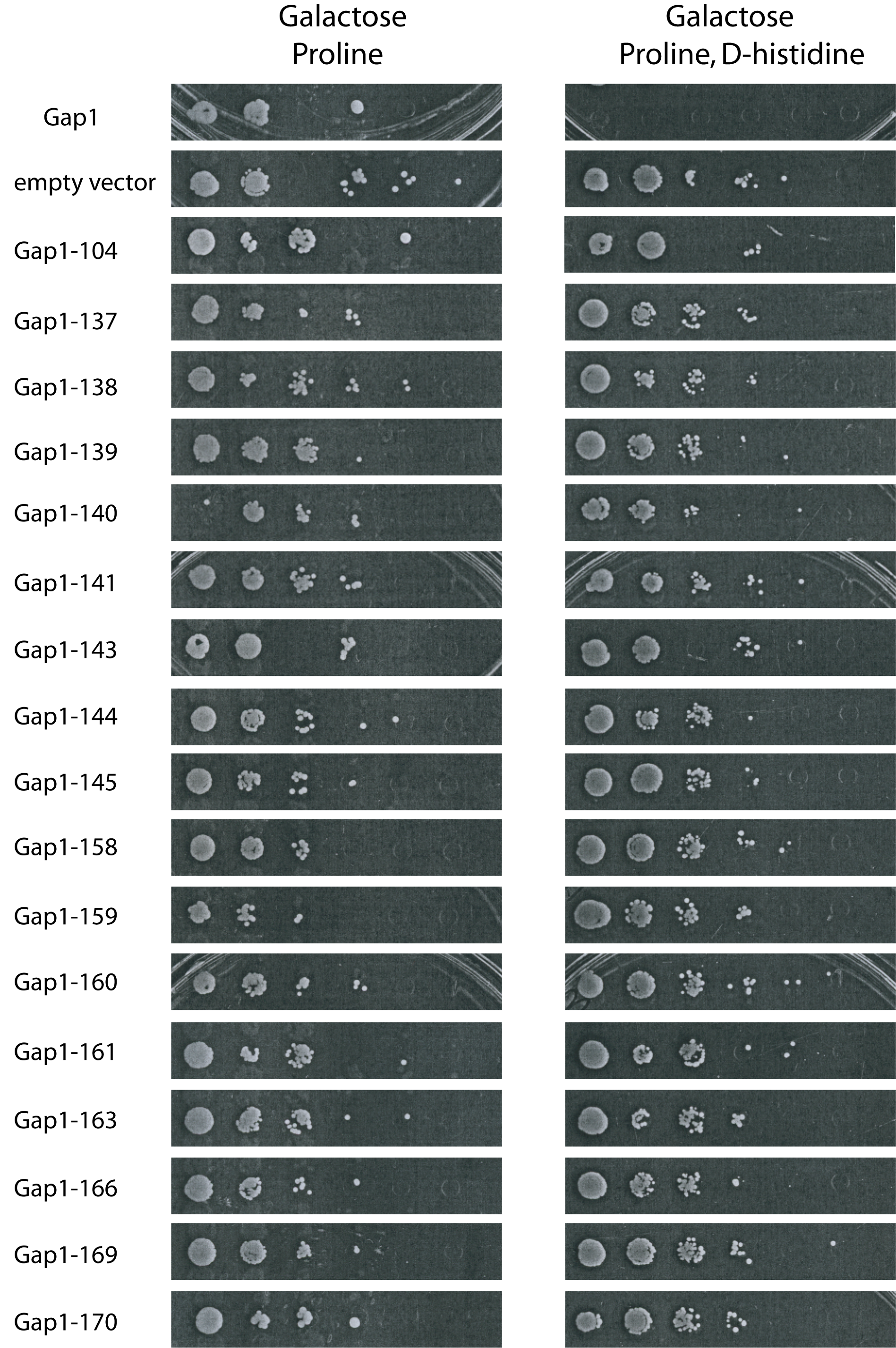

Supplement: Figure S4 — Overproduction of Shr3 does not suppress the growth phenotype of Gap1 mutants trapped in the ER. Strain ME042 (GAL1-SHR3 gap1Δ ura3) transformed with the pJOD10 (YCpGAL-GAP1-GFP) plasmid expressing the native permease (Gap1) or with equivalent plasmids expressing none Gap1 protein (empty vector), or one of the Gap1 mutants trapped in the ER (see text), were tested for growth on a solid medium containing galactose as a carbon source and proline as nitrogen source to which D-histidine was added or not. (TIF) [file pone.0018457.s004.tif]

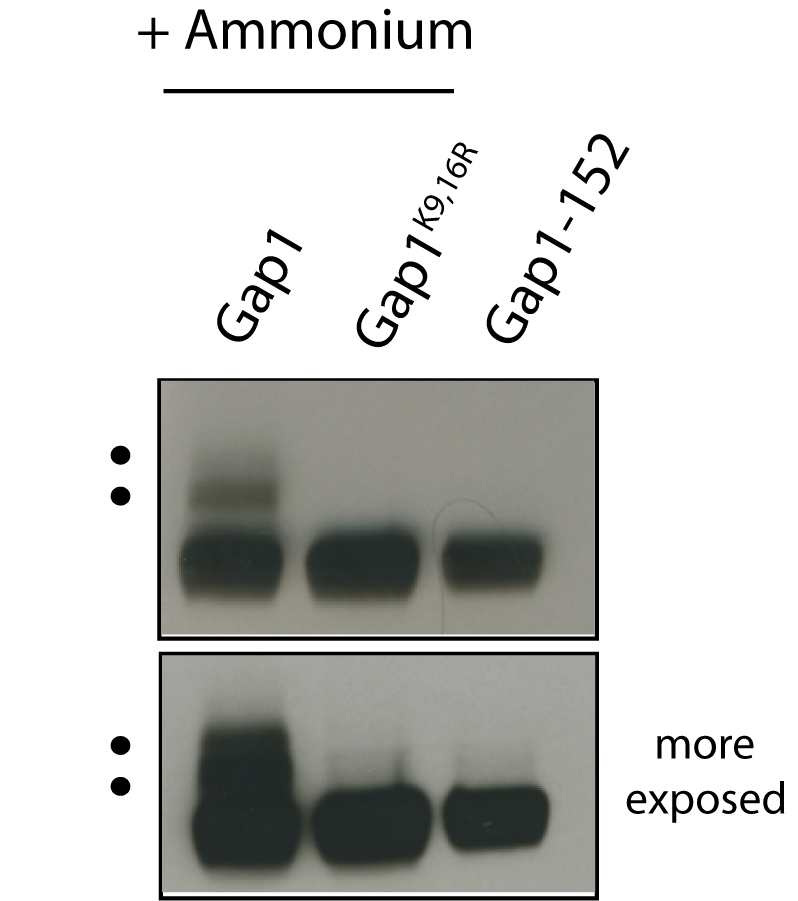

Supplement: Figure S5 — Gap1-152 does not seem to be ubiquitylated. Strain EL002 (gap1Δ vps27Δ ura3) transformed with the pJOD10 plasmid (Gap1) or with an equivalent plasmid encoding the Gap1K9,16R (pCJ038) or Gap1-152 (pNG18) variants was grown on galactose-proline medium. Ammonium (20 mM) was added for two hours to the cells expressing Gap1 or Gap1K9,16R. Cell extracts were then prepared and blotted with anti-GFP antibody. (TIF) [file pone.0018457.s005.tif]
