# Supplementary material for: Systematic Mutational Analysis of the Intracellular Regions of Yeast Gap1 Permease
Source: PLoS One. 2011 Apr 19;6(4):e18457. doi: 10.1371/journal.pone.0018457 (PMC3079708; doi:10.1371/journal.pone.0018457)
Supplement: Table S1 — Strains used in this study. (DOC) [file pone.0018457.s006.doc]

| **Table S1 - Strains used in this study** | | | | |
| --- | --- | --- | --- | --- |
| Strain |  | Genotype |  | Reference |
| EK008 |  | *gap1Δ ura3* |  | Laboratory collection |
| 32501d |  | *gap1Δ ssy1Δ ura3* |  | [1] |
| 30788b |  | *gap1Δ arg5,6 ura3* |  | This study |
| FB097 |  | *gap1Δ agp1Δ bap2Δ leu2Δ ura3* |  | [2] |
| EN121 |  | *gap1Δ end3Δ ura3* |  | [3] |
| EL002 |  | *gap1Δ vps27Δ ura3* |  | [4] |
| CJ005 |  | *gap1Δ npi1 ura3* |  | Laboratory collection |
| ME042 |  | *kanMX-GAL1p-SHR3 gap1∆ ura3* |  | This study |
|  |  |  |  |  |

References

1. Iraqui I, Vissers S, Bernard F, De Craene JO, Boles E, Urrestarazu A, André B (1999) Amino acid signaling in Saccharomyces cerevisie : a permease-like sensor of external amino acids and F-Box protein Grr1p are required for transcriptional induction of the AGP1 gene, which encodes a broad-specificity amino acid permease. Mol Cell Biol 19: 989-1001.

2. Bernard F, Andre B (2001) Genetic analysis of the signalling pathway activated by external amino acids in Saccharomyces cerevisiae. Mol Microbiol 41: 489-502.

3. Nikko E, Marini A-M, André B (2003) Permease recycling and ubiquitination status reveal a particular role for Bro1 in the multivesicular body pathway. J Biol Chem 278: 50732-50743.

4. Lauwers E, Jacob C, André B (2009) K63-linked ubiquitin chains as a specific signal for protein sorting into the multivesicular body pathway. Journal of Cell Biology 185: 493-502.
