# Supplementary material for: Systematic Mutational Analysis of the Intracellular Regions of Yeast Gap1 Permease
Source: PLoS One. 2011 Apr 19;6(4):e18457. doi: 10.1371/journal.pone.0018457 (PMC3079708; doi:10.1371/journal.pone.0018457)
Supplement: Table S2 — Plasmids used in this study. (DOC) [file pone.0018457.s007.doc]

| **Table S2. Plasmids used in this study** | | | | |
| --- | --- | --- | --- | --- |
| Plasmid |  | Description |  | Reference or source |
|  |  |  |  |  |
| pFL38 |  | *CEN-ARS URA3* |  | [5] |
| pJOD10 |  | *p416 GAL1-GAP1-GFP* |  | [3] |
| pCJ038 |  | *p416 GAL1-GAP1^K9,16R^-GFP* |  | Laboratory collection |
| pMA01 (Gap1-101) |  | *p416 GAL1-GAP1^SSY7-AAA^-GFP* |  | This study |
| pMA02 (Gap1-102) |  | *p416 GAL1-GAP1^FWC602-AAA^-GFP* |  | This study |
| pMA03 (Gap1-103) |  | *p416 GAL1-GAP1^T567A^-GFP* |  | This study |
| pMA10 (Gap1-113) |  | *p416 GAL1-GAP1^SNGS39-AAAA^-GFP* |  | This study |
| pMA12 (Gap1-115) |  | *p416 GAL1-GAP1^DETG47-AAAA^-GFP* |  | This study |
| pMA14 (Gap1-117) |  | *p416 GAL1-GAP1^WQDF55-AAAA^-GFP* |  | This study |
| pMA15 (Gap1-118) |  | *p416 GAL1-GAP1^KDSF59-AAAA^-GFP* |  | This study |
| pMA16 (Gap1-119) |  | *p416 GAL1-GAP1^KRVK63-AAAA^-GFP* |  | This study |
| pMA19 (Gap1-124) |  | *p416 GAL1-GAP1^ITAQ83-AAAA^-GFP* |  | This study |
| pMA21 (Gap1-125) |  | *p416 GAL1-GAP1^TPLK87-AAAA^-GFP* |  | This study |
| pMA22 (Gap1-128) |  | *p416 GAL1-GAP1^RIWN599-AAAA^-GFP* |  | This study |
| pMA23 (Gap1-129) |  | *p416 GAL1-GAP1^PRWY595-AAAA^-GFP* |  | This study |
| pMA27 (Gap1-130) |  | *p416 GAL1-GAP1^MATK591-AAAA^-GFP* |  | This study |
| pMA28 (Gap1-132) |  | *p416 GAL1-GAP1^EIAE583-AAAA^-GFP* |  | This study |
| pMA31 (Gap1-134) |  | *p416 GAL1-GAP1^VDLD575-AAAA^-GFP* |  | This study |
| pMA33 (Gap1-135) |  | *p416 GAL1-GAP1^GRRE571-AAAA^-GFP* |  | This study |
| pMA35 (Gap1-136) |  | *p416 GAL1-GAP1^AEKM563-AAAA^-GFP* |  | This study |
| pMA36 (Gap1-137) |  | *p416 GAL1-GAP1^LFIP559-AAAA^-GFP* |  | This study |
| pMA38 (Gap1-138) |  | *p416 GAL1-GAP1^RNWK555-AAAA^-GFP* |  | This study |
| pMA39 (Gap1-139) |  | *p416 GAL1-GAP1^KIYK551-AAAA^-GFP* |  | This study |
| pMA41 (Gap1-107) |  | *p416 GAL1-GAP1^PDNL15-AAAA^-GFP* |  | This study |
| pMA43 (Gap1-111) |  | *p416 GAL1-GAP1^TQEP31-AAAA^-GFP* |  | This study |
| pMA47 (Gap1-106) |  | *p416 GAL1-GAP1^EKNN11-AAAA^-GFP* |  | This study |
| pMA51 (Gap1-108) |  | *p416 GAL1-GAP1^KHNG19-AAAA^-GFP* |  | This study |
| pMA55 (Gap1-116) |  | *p416 GAL1-GAP1^SGSK51-AAAA^-GFP* |  | This study |
| pMA59 (Gap1-120) |  | *p416 GAL1-GAP1^PIEV67-AAAA^-GFP* |  | This study |
| pMA61 (Gap1-121) |  | *p416 GAL1-GAP1^DPNL71-AAAA^-GFP* |  | This study |
| pMA62 (Gap1-122) |  | *p416 GAL1-GAP1^SEAE75-AAAA^-GFP* |  | This study |
| pMA63 (Gap1-123) |  | *p416 GAL1-GAP1^KVAI79-AAAA^-GFP* |  | This study |
| pMA65 (Gap1-126) |  | *p416 GAL1-GAP1^HHLK91-AAAA^-GFP* |  | This study |
| pMA66 (Gap1-127) |  | *p416 GAL1-GAP1^NRH94-AAA^-GFP* |  | This study |
| pMA69 (Gap1-104) |  | *p416 GAL1-GAP1^DID566-AAA^-GFP* |  | This study |
| pMA70 (Gap1-136) |  | *p416 GAL1-GAP1^AEKM563-AAAA^-GFP* |  | This study |
| pMA71 (Gap1-114) |  | *p416 GAL1-GAP1^AVSI43-AAAA^-GFP* |  | This study |
| pMA74 (Gap1-112) |  | *p416 GAL1-GAP1^ITIP35-AAAA^-GFP* |  | This study |
| pMA79 (Gap1-133) |  | *p416 GAL1-GAP1^LLKQ579^-GFP* |  | This study |
| pMA85 (Gap1-131) |  | *p416 GAL1-GAP1^EKAI-AAAA^-GFP* |  | This study |
| pMA86 (Gap1-105) |  | *p416 GAL1-GAP1^SNT4-AAA^-GFP* |  | This study |
| pMA92 (Gap1-109) |  | *p416 GAL1-GAP1^ITID23-AAAA^-GFP* |  | This study |
| pMA93 (Gap1-110) |  | *p416 GAL1-GAP1^ESFL27-AAAA^-GFP* |  | This study |
| pMA116 (Gap1-143) |  | *p416 GAL1-GAP1^FTYY158-AAAA^-GFP* |  | This study |

| pNG01 (Gap1-140) | *p416 GAL1-GAP1^GELA146-AAA^-GFP* | This study |
| --- | --- | --- |
| pNG03 (Gap1-141) | *p416 GAL1-GAP1^VIFP150-AAAA^-GFP* | This study |
| pNG04 (Gap1-142) | *p416 GAL1-GAP1^ISGG154-AAAA^-GFP* | This study |
| pNG05 (Gap1-144) | *p416 GAL1-GAP1^ATRF162-AAAA^-GFP* | This study |
| pNG07 (Gap1-145) | *p416 GAL1-GAP1^IDE165-AAA^-GFP* | This study |
| pNG16 (Gap1-151) | *p416 GAL1-GAP1^SESV310-AAAA^-GFP* | This study |
| pNG18 (Gap1-152) | *p416 GAL1-GAP1^EPRK314-AAAA^-GFP* | This study |
| pNG20 (Gap1-153) | *p416 GAL1-GAP1^SVPK318-AAAA^-GFP* | This study |
| pNG29 (Gap1-157) | *p416 GAL1-GAP1^AEQR408-AAAA^-GFP* | This study |
| pNG30 (Gap1-158) | *p416 GAL1-GAP1^FLPE412-AAAA^-GFP* | This study |
| pNG32 (Gap1-159) | *p416 GAL1-GAP1^IFSY416-AAAA^-GFP* | This study |
| pNG34 (Gap1-160) | *p416 GAL1-GAP1^VDRK420-AAAA^-GFP* | This study |
| pNG36 (Gap1-161) | *p416 GAL1-GAP1^RFRK476-AAAA^-GFP* | This study |
| pNG38 (Gap1-162) | *p416 GAL1-GAP1^ALAA480-AAAA^-GFP* | This study |
| pNG40 (Gap1-163) | *p416 GAL1-GAP1^QGRG484-AAAA^-GFP* | This study |
| pNG41 (Gap1-164) | *p416 GAL1-GAP1^LDEL488-AAAA^-GFP* | This study |
| pNG42 (Gap1-165) | *p416 GAL1-GAP1^SFK491-AAA^-GFP* | This study |
| pNG45 (Gap1-166) | *p416 GAL1-GAP1^SFG168-AAA^-GFP* | This study |
| pNG47 (Gap1-167) | *p416 GAL1-GAP1^NMF221-AAA^-GFP* | This study |
| pNG49 (Gap1-168) | *p416 GAL1-GAP1^GVK224-AAA^-GFP* | This study |
| pNG51 (Gap1-169) | *p416 GAL1-GAP1^GRP423-AAA^-GFP* | This study |
| pNG53 (Gap1-170) | *p416 GAL1-GAP1^LVG426-AAA^-GFP* | This study |
| pNG55 (Gap1-171) | *p416 GAL1-GAP1^SPTG495-AAAA^-GFP* | This study |
| pMA142(Gap1-124 ^K9,16R^) | *p416 GAL1-GAP1^ITAQ83-AAAA./K9,16R^-GFP* | This study |
| pMA145(Gap1-152 ^K9,16R^) | *p416 GAL1-GAP1 ^EPRK314-AAAA./K9,16R^-GFP* | This study |
| pMA150(Gap1 ^9KR^) | *p416 GAL1-GAP^/9KR^-GFP* | This study |
| pMA151(Gap1-152 ^9KR^) | *p416 GAL1-GAP ^EPRK314-AAAA./9KR^-GFP* | This study |

References

1. Iraqui I, Vissers S, Bernard F, De Craene JO, Boles E, Urrestarazu A, André B (1999) Amino acid signaling in Saccharomyces cerevisie : a permease-like sensor of external amino acids and F-Box protein Grr1p are required for transcriptional induction of the AGP1 gene, which encodes a broad-specificity amino acid permease. Mol Cell Biol 19: 989-1001.

2. Bernard F, Andre B (2001) Genetic analysis of the signalling pathway activated by external amino acids in Saccharomyces cerevisiae. Mol Microbiol 41: 489-502.

3. Nikko E, Marini A-M, André B (2003) Permease recycling and ubiquitination status reveal a particular role for Bro1 in the multivesicular body pathway. J Biol Chem 278: 50732-50743.

4. Lauwers E, Jacob C, André B (2009) K63-linked ubiquitin chains as a specific signal for protein sorting into the multivesicular body pathway. Journal of Cell Biology 185: 493-502.

5. Bonneaud N, Ozier-Kalogeropoulos O, Li GY, Labouesse M, Minvielle-Sebastia L, Lacroute F (1991) A family of low and high copy replicative, integrative and single-stranded S. cerevisiae/E. coli shuttle vectors. Yeast 7: 609-615.
